# Perimyocarditis With Acute Heart Failure as the First Manifestation of Systemic Lupus Erythematosus

**DOI:** 10.7759/cureus.26707

**Published:** 2022-07-09

**Authors:** Juan Camilo Santacruz, Marta Juliana Mantilla, Igor Rueda, Gustavo Rodríguez-Salas, Sandra Pulido, John Londono

**Affiliations:** 1 Spondyloarthropathies Research Group, Universidad de La Sabana, Chía, COL; 2 Rheumatology Department, Universidad Militar Nueva Granada, Bogotá, COL

**Keywords:** systemic lupus erythematosus, glucocorticoids, libman-sacks endocarditis, acute heart failure, myopericarditis

## Abstract

Cardiac abnormalities are common in patients with systemic lupus erythematosus (SLE). However, many of them tend to be mild or asymptomatic and can be recognized by non-invasive studies such as transthoracic echocardiography and cardiac magnetic resonance imaging (CMR). However, heart failure secondary to perimyocarditis as the initial manifestation of SLE remains an extremely rare form of presentation. Below, we present the case of an adult female patient who initially consulted due to symptoms of acute dyspnea, atypical chest pain, and edema of the lower limbs, who underwent a chest X-ray as part of the initial studies, which described an increase in the cardiac silhouette associated with diffuse opacities in both lung fields. The admission electrocardiogram only showed sinus tachycardia and nonspecific alterations of the T wave, with an initial report of frankly elevated cardiac biomarkers compatible with acute myocardial injury together with the positivity of specific antibodies for SLE.

## Introduction

Systemic lupus erythematosus (SLE) is a systemic autoimmune disease of variable severity with a tendency to present flares during its evolution [1]. Tissue damage attributed to the disease is caused by autoantibodies or immune complex deposition found primarily in the kidneys, heart, blood vessels, central nervous system, skin, lungs, muscles, and joints, which leads to significant morbidity and increased mortality [2]. Cardiac involvement is the second most frequent organic manifestation of SLE, followed by lupus nephritis, being diagnosed in almost 50% of patients. Pericarditis is the most prevalent cardiac manifestation, presenting in 30 to 50% of patients during the course of the disease. However, pericardial involvement with severe myocardial dysfunction occurs infrequently [3]. Valvular disease, unlike pericardial involvement, is one of the most prevalent and important forms of cardiac involvement in patients with SLE, representing a frequent cause of morbidity [4]. Pericarditis, like other types of serositis, occurs more frequently when SLE is active in other organs [5]. Lupus myocarditis can present clinically with symptoms of heart failure that include tachycardia and dyspnea, but can also present chest discomfort, fever and/or myopericarditis, although its presentation is usually asymptomatic or subclinical [6]. Cardiac magnetic resonance imaging (CMR) has been very useful for the study of several cardiomyopathies, representing several highly specific patterns of myocardial damage [7]. Cardiac involvement in immune-mediated systemic diseases can be evaluated by characterizing the tissue by CMR with T1 and T2-weighted images and late gadolinium enhancement, achieving a better view of myocardial contractility, the presence of intracardiac masses, fibrosis, ischemia, pericardial and aortic involvement, while transesophageal echocardiography is the most useful method to detect valvular disease [8].

## Case presentation

**Figure 1:**
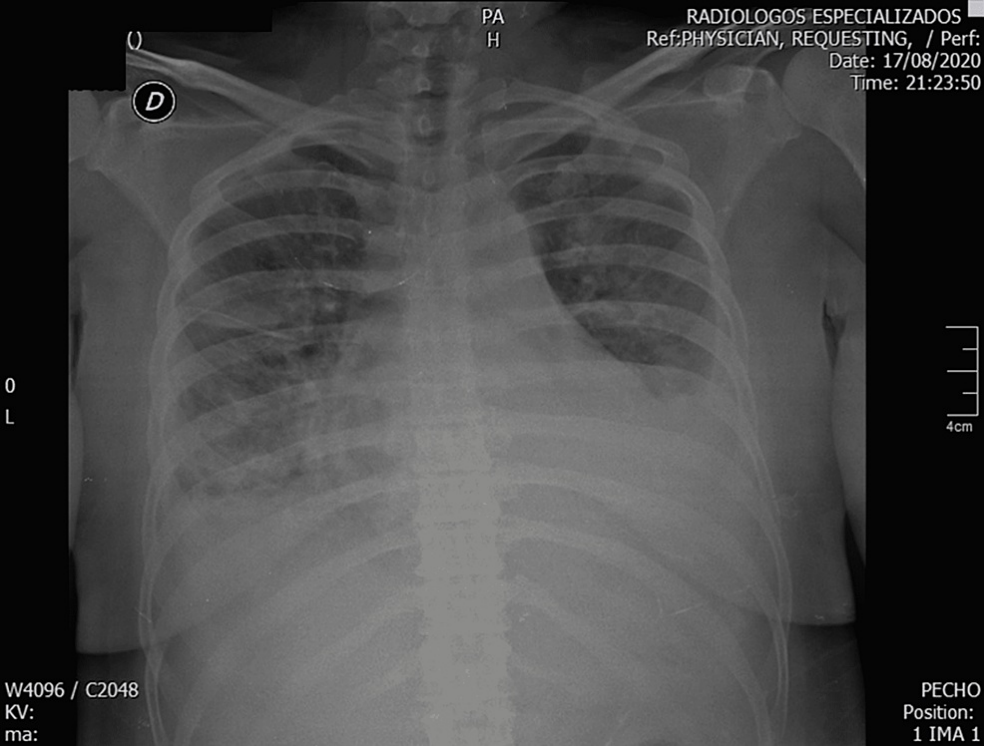
Chest X-ray Cardiac silhouette increased in size, a widening of the upper mediastinum of probable vascular origin is shown, and the costodiaphragmatic angles are blurred by pleural effusion, predominantly on the left side. Multiple diffuse distribution opacities with a tendency to bibasal consolidation are observed in the lung fields.

This is a 40-year-old female patient with a history of primary hypothyroidism controlled with low doses of levothyroxine, who was admitted to the emergency department due to symptoms of acute dyspnea associated with chest pain, located in the left hemithorax, stabbing type, of moderate intensity, persistent, which limited his functional class for three weeks. Physical examination on admission revealed the presence of tachycardia, tachypnea, use of accessory muscles, coarse rales in both lung fields, and lower limb edema, suggestive of acute heart failure. The possibility of pneumonia due to COVID-19 was considered as the initial diagnosis given that the admission X-ray described bilateral diffuse opacities with a tendency to consolidate in the lung bases, increased cardiac silhouette and widening of the superior mediastinum, completing 10 days of dexamethasone treatment (Figure 1).

**Figure 2:**
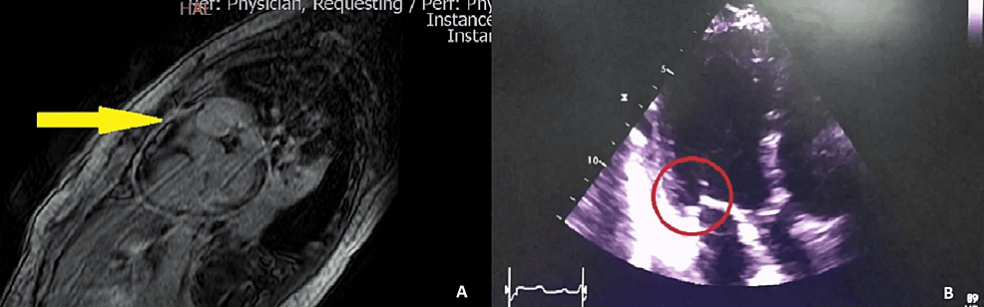
Cardiac magnetic resonance and transthoracic echocardiogram Panel A shows that the two ventricles are dilated, hypokinetic, with severe systolic dysfunction of the left ventricle. Pericardial thickening is observed adjacent to the mid-apical inferolateral segments of 3mm with enhancement after injecting contrast medium. Myocarditis is established by the Lake Louise criteria showing areas of high signal intensity in a non-ischemic distribution pattern on late gadolinium enhancement images in T1 and high signal intensity of the pericardium consistent with pericardial inflammation. Panel B shows an echodense, sessile mass, adhered to the posterior wall of the left atrium 7mm from the mitral annulus, compatible with vegetation.

However, she had a negative reverse transcription-polymerase chain reaction report for COVID-19 and a negative serological blood test for Chagas disease. The admission electrocardiogram only showed sinus tachycardia and nonspecific changes in the T wave. She subsequently develops hypoxemic respiratory failure requiring invasive ventilatory support for which she is transferred to the intensive unit care (IUC). During her stay in the IUC, she presented acute kidney injury with dialysis urgency criteria, requiring renal replacement therapy. Once hemodynamic stability is achieved after hemodialysis, an echocardiogram is performed showing a severe compromise of the left ventricular ejection fraction (LVEF) (29%) together with the description of an echodense, sessile mass, adhered to the posterior wall of the left atrium, 7mm of the mitral annulus, compatible with a thrombus, vegetation, or tumor, requiring its complementary study with a CMR that showed pericardial thickening and late gadolinium enhancement compatible with acute pericarditis (Figure 2).

The mass described in the initial echocardiogram was not visualized on CMR probably due to glucocorticoid treatment. The report of quantitative high-sensitivity troponin on admission was frankly positive, finally considering the diagnosis of perimyocarditis. Subsequently, the studies were expanded to rule out infectious or autoimmune causes of myocardial involvement, considering an autoimmune etiology more likely given the presence of proteinuria and active urinary sediment in the admission urinalysis. The results of the immunology laboratories showed high titers of antinuclear antibodies (ANA) (1:2560 homogeneous pattern), anti-dsDNA, anti-Ro, anti-RNP and anti-SM together with a marked consumption of complement, attributing SLE as the main cause of cardiac involvement. The patient required methylprednisolone pulses (1 gram intravenously per day for three days) with a subsequent transition to prednisolone at a dose of 1 mg/kg/day and cyclophosphamide (1 g/m2 in monthly pulses) in the context of rapidly progressive glomerulonephritis type 2, achieving extubation and clinical recovery with normalization of the LVEF. Below is a table with the most representative paraclinical tests since admission to the emergency department (Table 1).

**Table 1:** Most representative laboratory findings of clinical course and reference values ANA: antinuclear antibodies; Anti-HBc Ab: hepatitis B core total antibodies; Anti-HCV: Anti-Hepatitis C Antibodies; CRP: C-reactive protein; HBsAg: hepatitis B surface antigen; RT-PCR: reverse transcription-polymerase chain reaction; VDRL: Venereal Disease Research Laboratory

Laboratories	Result	Reference values
Total white blood cell count (/µl)	2590	4000 - 10,000
Total neutrophil count (/µl)	5350	2000 - 7500
Total lymphocyte count (/µl)	2870	1500 - 4000
Total eosinophils count (/µl)	150	40 - 400
Total platelet count (/µl)	607,000	150,000 - 400,000
Hemoglobin (gr/dL)	6.3	13.5 - 17.5
Hematocrit (%)	19.2	35 - 47
Mean Corpuscular Volume (fl)	74.1	80 - 100
Sodium (meq/L)	127.9	135 - 148
Potassium (meq/L)	6.54	3.5 - 5
Creatinine (mg/dL)	5.49	0.51 - 0.95
Ureic nitrogen (mg/dL)	51.5	6 - 20
Glycemia (mg/dL)	128	74 - 106
Lactate dehydrogenase (UI/L)	273	135 - 214
Ferritin (mg/mL)	1784	13 - 150
CRP (mg/L)	145.1	0.6-5
RT-PCR for COVID-19	negative	-
High sensitivity troponin T (ng/mL)	0.055	less than 0.014
High sensitivity troponin T control (ng/mL)	0.0978	less than 0.014
ANA (titers)	Positive 1:2560	less than 1:80
Anti-RNP antibodies (U)	130.7	less than 20
Anti-SM	88.4	less than 20
Anti-Ro	87.1	less than 20
Anti-La	7.19	less than 20
Anti-DNA (IU/mL)	Positive 1:160	less than 1:10
C3 (mg/dL)	48.8	88 - 201
C4 (mg/dL)	6.6	15 - 45
VDRL test	non-reactive	-
HBSAg	non-reactive	-
Anti-HBc Ab	non-reactive	-
Anti-HCV	non-reactive	-
Urinalysis	Red blood cells casts - Proteinuria	-
24-hour urine protein measurement (mg)	400.7	less than 140

## Discussion

Heart disease is common among patients with SLE, manifesting as either valvular or pericardial disease, myocardial dysfunction, or coronary artery disease secondary to accelerated atherosclerosis [9]. Within this group, pericarditis is the most frequent cardiac manifestation. Approximately 25% of all patients with SLE develop symptomatic pericarditis at some point during the course of the disease, most frequently with associated pleurisy. However, autopsy studies reveal a higher rate of subclinical pericarditis, and it is unusual for pericarditis to be the only presenting symptom of the disease [10]. Patients with lupus pericarditis typically present with tachycardia, substernal or precordial chest discomfort, dyspnea, and positional pain, these findings being similar to patients with pericarditis without SLE. Pericardial effusion can occur at any time in more than 50% of patients, and pericarditis can precede the clinical signs of SLE [11]. Pericardial fluid from patients with lupus pericarditis typically reveals an inflammatory exudate with a predominance of neutrophils. Pericardial biopsy is not necessary to establish its diagnosis, but if histopathology is performed, it usually shows mononuclear cells, fibrinous material, and immune complex deposits [12]. Acute lupus myocarditis as a presenting symptom of SLE continues to be a rare entity and only 0.37% of cases debut with acute heart failure [13]. Global or patchy hypokinesia, without a specific coronary artery distribution, may be an echocardiographic indication of myocarditis and is present in approximately 5 to 20% of patients [14]. Regarding the estimates of the prevalence of Libman-Sacks endocarditis in patients with SLE, they range between 11% and 74%. Libman-Sacks endocarditis most commonly affects the mitral valve, although any valve can be affected [15]. Clinically significant valve regurgitation and/or stenosis is rare and surgical treatment is required in only 4-6% of patients [16]. There is a 3.5-fold increased risk of valvular heart disease in patients with positive antiphospholipid antibodies and Libman-Sacks endocarditis, particularly with positive lupus anticoagulant and anticardiolipin IgG antibodies, since these antibodies have been identified in the center of lesions valves along with the deposition of immune complexes [17]. Pericarditis is often a manifestation of an SLE flare, so treatment is based on a short course of nonsteroidal anti-inflammatory drugs (NSAIDs), a medium or low dose of glucocorticoids, and the start of hydroxychloroquine. The treatment of lupus myocarditis has not been evaluated in controlled clinical trials, but the guidelines recommended by experts suggest the initiation of high-dose glucocorticoids (Methylprednisolone 1 gram intravenously daily for three days), cyclophosphamide, or mycophenolate mofetil as first-line therapy [18]. There are some reports that indicate the benefit of intravenous immunoglobulin with improvement in the LVEF, and in the case of severe or refractory myocarditis, rituximab has been used successfully as additional therapy [19]. Treatment of Libman-Sacks endocarditis is indefinite anticoagulation with therapeutic doses of low molecular weight heparin due to the risk of stroke or embolization to other organs. The use of high-dose glucocorticoids has been considered when vegetation is identified at an early, active stage, but the evidence for this treatment with respect to size reduction or resolution is still inconclusive [20].

## Conclusions

Perimyocarditis and acute heart failure are unusual presentations of SLE. The diagnostic approach to perimyocarditis is the same as for myocarditis, requiring high doses of glucocorticoids and cyclophosphamide, generally with good clinical results. Pericarditis and myocarditis reflect high SLE activity, so looking for another organic compromise can support the diagnosis. Although it is true that high doses of glucocorticoids have not been able to demonstrate the reduction or resolution of the vegetation in Libman-Sacks endocarditis, it may be plausible given that part of the valvular heart involvement is based on the deposition of immune complexes.
